# Attenuated Amiloride-Sensitive Current and Augmented Calcium-Activated Chloride Current in Marsh Rice Rat (*Oryzomys palustris*) Airways

**DOI:** 10.1016/j.isci.2019.08.011

**Published:** 2019-08-08

**Authors:** Shin-Ping Kuan, Yan-Shin J. Liao, Katelyn M. Davis, Jonathan G. Messer, Jasenka Zubcevic, J. Ignacio Aguirre, Leah R. Reznikov

**Affiliations:** 1 Department of Physiological Sciences, University of Florida, Gainesville, FL 32610, USA

**Keywords:** Bio-Electrochemistry: Physiology, Molecular Physiology

## Abstract

Prolonged heat and sea salt aerosols pose a challenge for the mammalian airway, placing the protective airway surface liquid (ASL) at risk for desiccation. Thus, mammals inhabiting salt marshes might have acquired adaptations for ASL regulation. We studied the airways of the rice rat, a rodent that inhabits salt marshes. We discovered negligible Na^+^ transport through the epithelial sodium channel (ENaC). In contrast, carbachol induced a large Cl^−^ secretory current that was blocked by the calcium-activated chloride channel (CaCC) inhibitor CaCCinhi-A01. Decreased mRNA expression of α, β, and γ ENaC, and increased mRNA expression of the CaCC transmembrane member 16A, distinguished the rice rat airway. Rice rat airway cultures also secreted fluid in response to carbachol and displayed an exaggerated expansion of the ASL volume when challenged with 3.5% NaCl. These data suggest that the rice rat airway might possess unique ion transport adaptations to facilitate survival in the salt marsh environment.

## Highlights

• Rice rat airway cultures show limited epithelial sodium channel activity
• mRNA expression levels of epithelial sodium channel were decreased
• Exaggerated calcium-activated chloride channel activity was also observed
• Rice rat airway fluid secretion properties were unique compared to other mammals

## Graphical Abstract

**Figure:**
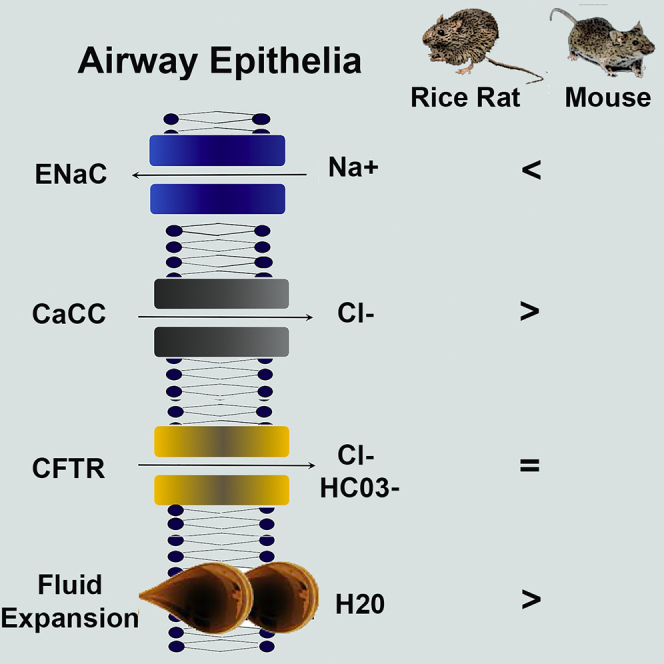

## Introduction

The airway epithelium is a critical barrier to the external environment and is protected by a thin layer of fluid known as the airway surface liquid (ASL). ASL facilitates the entrapment of particles and bacteria, prevents airway desiccation, humidifies inspired air, and supports mucociliary transport (Widdicombe, 1997). ASL volume and composition are regulated by active and passive epithelial cell ion transport that is in part dependent on the apical epithelial sodium channel (ENaC) (Chambers et al., 2007), the cystic fibrosis transmembrane conductance regulator (CFTR), and the calcium (Ca^2+^)-activated Cl^−^ channels (CaCC) (Frizzell and Hanrahan, 2012).

The importance of these proteins in regulating ASL composition and volume has been repeatedly shown. For example, in mice genetically modified to hyperabsorb Na^+^ through overexpression of the β ENaC subunit, ASL is depleted (Mall et al., 2004), whereas mice lacking the α ENaC subunit die shortly after birth due to an inability to clear fluid from their lungs (Hummler et al., 1996). Mutations in CFTR cause the life-shortening autosomal recessive disorder, cystic fibrosis (CF) (Riordan et al., 1989). The loss of CFTR-mediated Cl^−^ and bicarbonate secretion in CF sets up an airway environment prone to infection, inflammation, mucous obstruction, ASL depletion, and impaired fluid secretion (Boucher, 2007, Pezzulo et al., 2012). CaCC-mediated Cl^−^ transport also regulates ASL volume acutely (Tarran et al., 2002), and in mice lacking the CaCC, TMEM16A, there is defective fluid secretion (Rock et al., 2009).

Salt marshes are coastal wetlands characterized by high salt concentrations, intense heat, and periods of flooding (Silliman, 2014). The increased salinity and extreme temperatures pose a challenge for the mammalian airway. First, the dissipation of heat through panting (e.g., mouth breathing) can cause evaporative loss of water from the ASL lining the trachea and bronchi (Widdicombe, 1997). Second, inhalation of hypertonic solutions, such as those encountered in atmospheric sea salt aerosols (Gabriel et al., 2002), can modify ASL composition. Both these events can acutely increase ASL tonicity. The airway responds to hypertonic solutions via two major mechanisms. First, hypertonic solutions osmotically draw water onto the airway surface (Chambers et al., 2007, Harvey et al., 2011). Second, hypertonic solutions activate the vagus nerve, causing the release of acetylcholine from nerve terminals innervating the airway (Wine, 2007). Acetylcholine then binds to muscarinic receptors located on epithelial cells, leading to increased intracellular Ca^2+^ and fluid secretion that is dependent on activation of CaCC (Caputo et al., 2008, Catalan et al., 2015, Frizzell and Hanrahan, 2012).

The rice rat (*Oryzomys palustris*) is a medium-sized nocturnal rodent (40–80 g) from the family Cricetidae. It can be found inhabiting the salt marshes of North and South America (Hamilton, 1946), where it is well adapted for swimming (Wolfe and Esher, 1981). In captivity, the rice rat adapts well to standard laboratory conditions and requires minimal modifications (Aguirre et al., 2015).

In the current study, we hypothesized that the rice rat airway might have developed unique adaptations to ensure survival in the harsh salt marsh environment. Specifically, we hypothesized that exposure to high temperatures and sea salt aerosols might have resulted in an evolutionary pressure that decreased Na^+^ absorption through ENaC and increased dependence upon CaCC for Cl^−^ transport. To test this hypothesis, we examined net ion transport with Ussing chambers (Chen et al., 2010, Ostedgaard et al., 2011, Stoltz et al., 2013) and assessed fluid secretion in response to cholinergic stimulation and hypertonic solutions using reflective light microscopy (Harvey et al., 2011). Expression levels of key ion channels were also quantified. As controls, we utilized tracheal cultures of the mouse and the pig, animals that do not normally inhabit salt marshes and whose airway electrophysiological properties are well characterized (Chen et al., 2010, Ostedgaard et al., 2011, Rogers et al., 2008, Stoltz et al., 2013).

## Results

### Cultured Rice Rat Airways Show Exaggerated CaCC-Mediated Short Circuit Current, but Negligible Basal Amiloride-Sensitive Short Circuit Current

**Figure 1:**
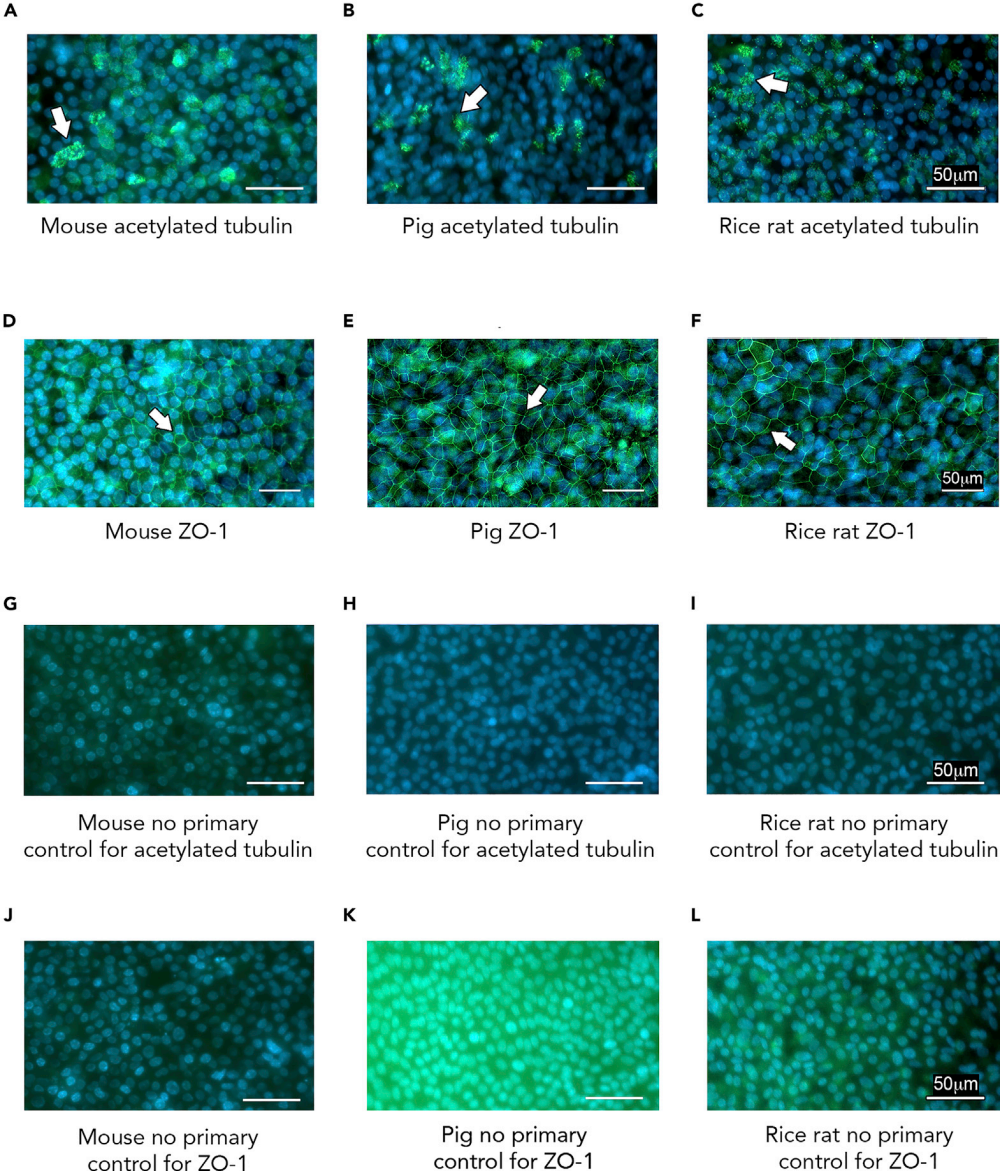
Tight Junctions and Cilia in Rice Rat Cultures (A–L) *En face* images of tracheal cultures of mouse (A), piglet (B), and rice rat (C) showing expression of the cilia protein acetylated alpha tubulin. *En face* images of tracheal cultures of mouse (D), piglet (E), and rice rat (F) showing expression of the tight junction protein ZO-1. *En face* images of tracheal cultures of mouse (G), piglet (H), and rice rat (I) incubated with only the goat anti-mouse 488 secondary antibody (secondary used for the detection of acetylated tubulin). *En face* images of tracheal cultures of mouse (J), piglet (K), and rice rat (L) incubated with only goat anti-rabbit 488 secondary antibody; this secondary was used for the detection of ZO-1. ZO-1, zonula occludens protein 1. Hoechst was used to detect nuclei. For all panels, scale bars, 50 μm. Antibody detection of ZO-1 and acetylated alpha tubulin was performed on two separate occasions for each species. White arrows show example of specific staining.

We cultured primary tracheal airway epithelia from the rice rat, mouse, and pig at the air-liquid interface. We first confirmed the presence of key features of airway epithelia, including cilia (Jain et al., 2010) (Figures 1A–1C) and expression of the tight junction protein zonula occludens protein-1 (ZO-1) (Vermeer et al., 2009) (Figures 1D–1F). No staining was observed for either ZO-1 or acetylated alpha tubulin in negative controls (Figures 1G–1L), suggesting specific detection.

**Figure 2:**
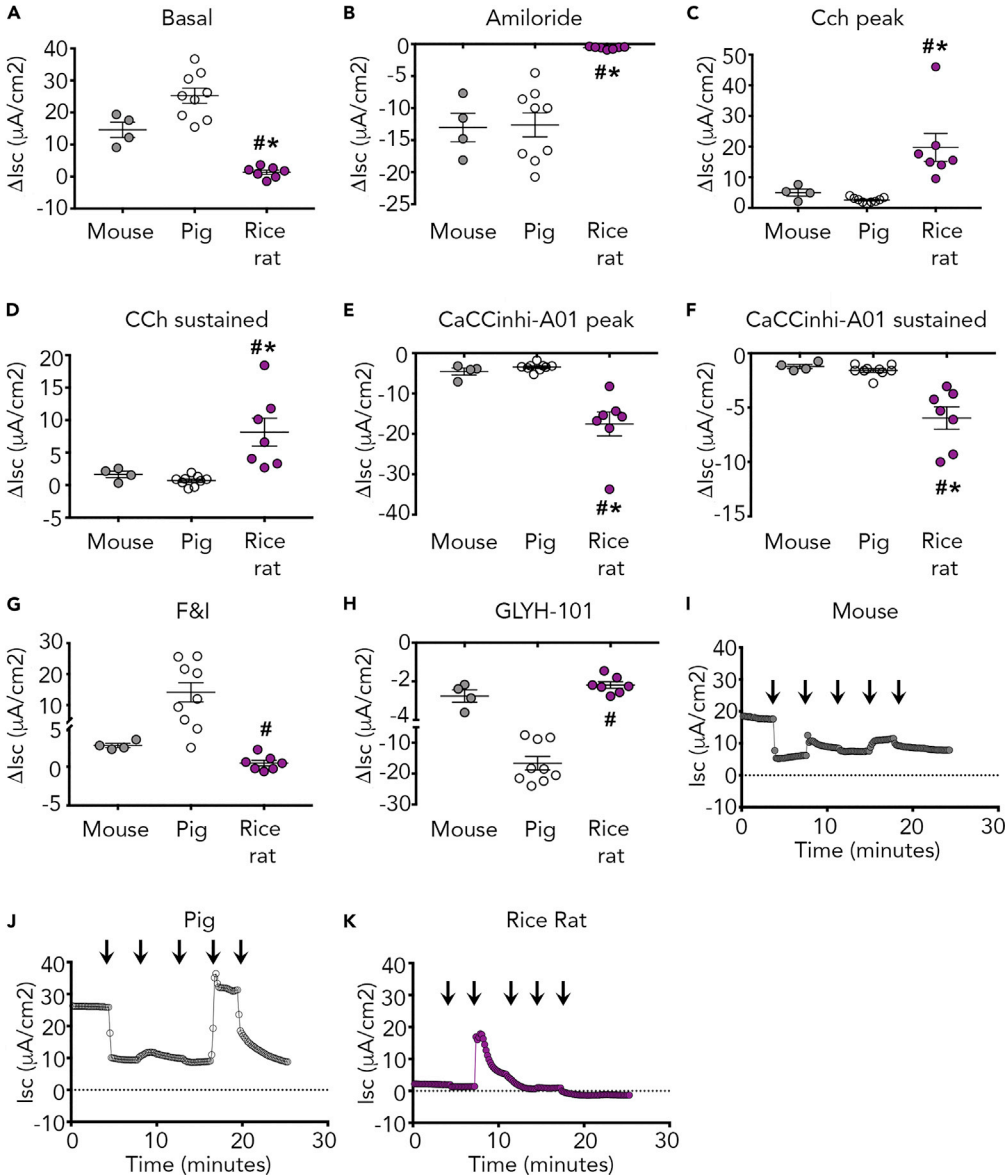
Primary Tracheal Cultures of Rice Rats Have Limited Basal and Amiloride-Sensitive Current, but Large Carbachol-Mediated Cl^−^ Secretion (A–H) Basal short circuit current (Isc) measurements in primary cultures of rice rat and piglet (A). Averaged delta (Δ) Isc measurements shown for 100 μM apical amiloride (AMIL) (B), 100 μM basolateral carbachol (CCh) (peak and sustained responses in C and D, respectively), 30 μM apical CaCCinhi-A01for inhibition of peak (E) and sustained (F) CCh-mediated current, apical 10 μM forskolin and 100 μM IBMX (F&I) (G), and 100 μM apical GLYH-101 (H). (I–K) Representative short circuit current (Isc) trace in mouse (I), piglet (J), and rice rat (K) airway cultures. Arrows indicate addition of drugs (as described in Methods). CCh, carbachol; F&I, forskolin and IBMX. For all panels, n = 4 cultures from mice (2 female cultures and 2 male cultures representing 6 female and 6 male subjects); n = 7 cultures from the rice rat (4 female cultures and 3 male cultures representing 8 female and 6 male subjects); n = 9 porcine cultures (5 female and 4 male representing 5 individual female and 4 individual male subjects). ∗p < 0.05 compared with mouse; ^#^p < 0.05 compared with pig. All data are shown as mean ± SEM.

We next examined net ion transport. We found that the rice rat tracheal cultures displayed minimal basal short circuit current (Figures 2A and 2I). This basal value was significantly lower than the basal short circuit current measured in either the piglet or the mouse (Figures 2A and 2I–2K). We also found minimal amiloride-sensitive Isc in the rice rat tracheal cultures compared with the mouse or pig (Figures 2B and 2I–2K). These data suggested that the rice rat had diminished Na^+^ transport through ENaC.

We next stimulated epithelium with carbachol basolaterally to activate CaCC (Drumm et al., 1991). Carbachol induced a large transient outward current (Figure 2C) in rice rat cultures. Both the peak and sustained responses to carbachol were significantly greater in the rice rat compared with the mouse or pig (Figures 2C, 2D, and 2I–2K). Application of CaCCinhi-A01 inhibited carbachol-induced Cl^−^ secretion to a greater extent in the rice rat (Figures 2E, 2F, and 2I–2K). Thus, rice rat airways displayed augmented CaCC-mediated Cl^−^ secretion compared with pigs and mice.

We applied forskolin and 3-isobutyl-1-methylxanthine (IBMX) (F&I) to activate CFTR. We found significantly less F&I-mediated Cl^−^ secretion in the rice rat compared with the pig, but not compared with the mouse (Figures 2G and 2I–2K). Application of the CFTR inhibitor GlyH-101 caused a greater inhibition of F&I-mediated current in the pig compared with the rice rat (Figures 2H, 2J, and 2K). The amount of GlyH-101-inhibited current in the rice rat and mouse was not different (Figures 2H, 2I, and 2K). These data suggested that the rice rat had similar CFTR activity as the mouse, but comparatively less than the pig.

### Rice Rat Airway Cultures Have Decreased Expression of ENaC and Increased Expression of TMEM16a

Our ion transport studies suggested alterations in the transport mediated by ENaC and CaCC. Given that the rice rat genome is unknown, the tools available for quantifying ion channel expression at either the transcript or the protein level are limited. To circumvent this, we cloned small segments of mRNA for rice rat CFTR, TMEM16A, α ENaC, β ENaC, γ ENaC, CHRM3, and RPL13a (Table 1). Based on those sequences, we then designed “universal” primers to allow for direct comparisons between mouse and rice rat using the same primer set (Table 2). RPL13a was used as a housekeeping control (Shah et al., 2016). We preferentially focused on the mouse and rice rat because they are more closely related phylogenetically.

**Table 1:** Primers Used for Cloning Small Coding Regions of Rice Rat Genes

Gene	Forward Primer	Reverse Primer
*CFTR*	CAA CAC AGT TCT TGG AGA AGG TGG AG	TCT ACT GAG AAC CTG CGT AAG GTC TC
*TMEM16A*	GCT GCC ACT TTC ATG GAG CAC T	GTT CAT GGC CAA GGC TGC AG
*CHRM3*	TCG CCT TTG TTT CCC AAC ATC AG	CCA GCA GAT TCA TGA CAG AAG CAT T
*SCNN1a*	ATG CGG GCA CCG TCA C	TCG AAC AGC AAA GCA AAC TGC C
*SCNN1b*	ATG CCA GTG AAG AAG CTC CTG A	AGC AAG TGC TTG ACC TTG GAG T
*SCNN1g*	ATG GCG CCT GGA GAG AAG ATC	TGT TTT GTC TCA CTG TCC AAG TCA G
*RPL13A*	TTC TGG CGC ACT GTG CGA G	TTG GTC TTG AGG ACC TCT GTG AAC
*NR3C1*	GAG TCC TTG GAG GTC AGA CCT	CCA ACA GGA ATT GGT GGA ATG ACA
*NR3C2*	GTT AAG AGC CCA ATC ATC TGT CAT G	GTG TTG GAA GGG CTG GAA A

**Table 2:** Primers Used for Quantitative RT-PCR

Gene	Forward Primer	Reverse Primer
*CFTR*	TGT AAA TTG ATG GCC AAC AAA A	GAA CTG AAG TCT GGA CGT AGA CTT T
*TMEM16A*	CAT GGA GCA CTG GAA ACG GAA G	CCT GCC ATG GCT GTC CTA AC
*CHRM3*	TCT GGC AAG TGG TCT TCA TTG C	GAA ATG ACC CCG ATG ATC AGA TCT G
*SCNN1a*	GAT CGA GTT CCA CCG CTC CTA	CCC AGA AGG CCG TCT TCA TG
*SCNN1b*	GAG GGG CCC AAG AAG AAG	TGA AGC CCA TGG AGA GCG AG
*SCNN1g*	GCA TCG TGG TGT CCC GA	CAC TGT ACT TGT AGG GGT TGA TAT TGC
*RPL13A*	CGC CTC AAG GTG TTG GAT G	GTC ACT GCC TGG TAC TTC CA
*NR3C1*	CAC TGC CCC AAG TGA AAA CAG A	TCC AGA GGT ACT CAC GCC ATG
*NR3C2*	GCA TCA ACT CCA TGT CCT CCT	GCT GCT TAA TGG ACT TGA AAG AGG

**Figure 3:**
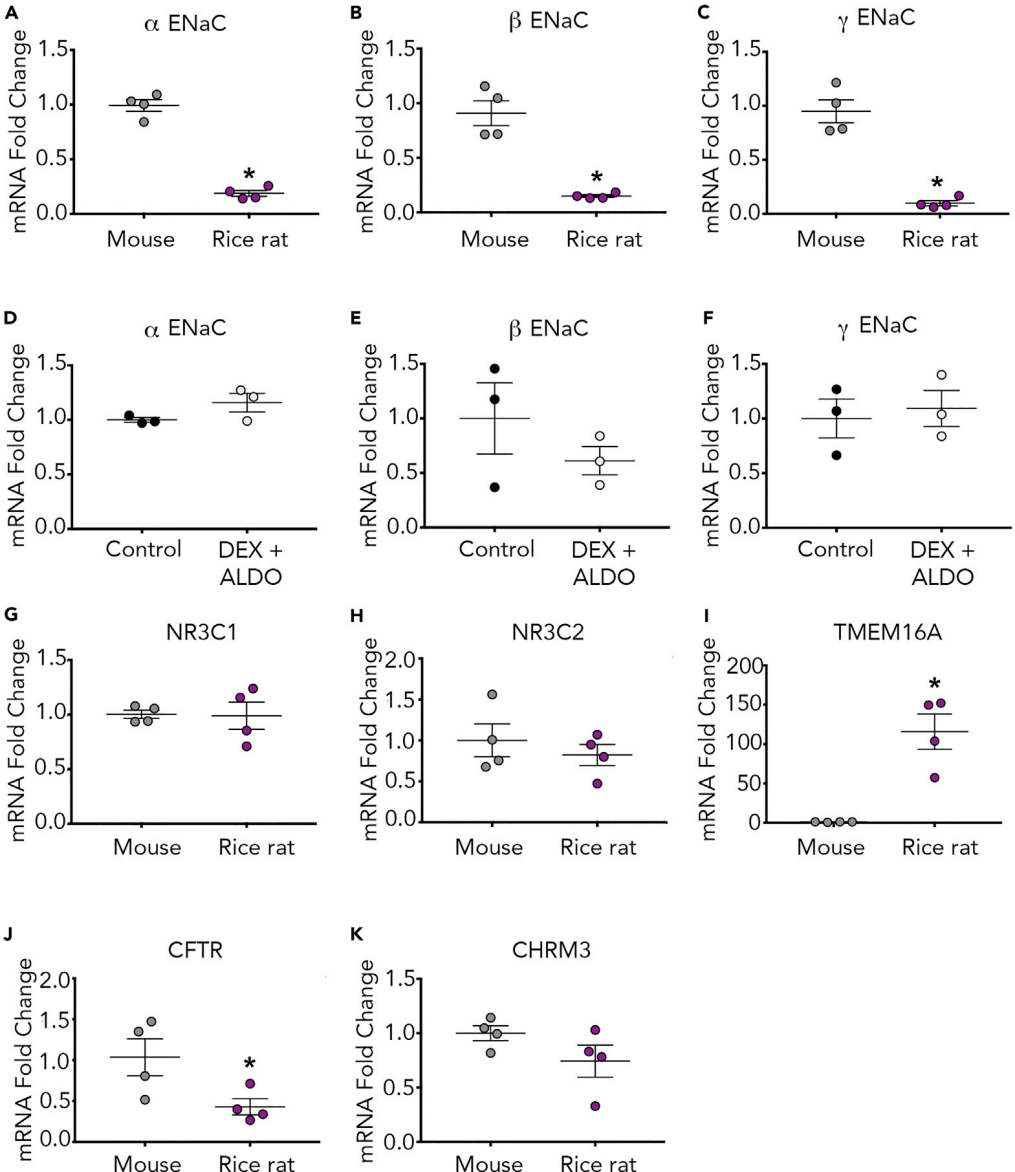
Rice Rat Tracheal Cultures have Decreased ENaC Expression and Increased TMEM16A Expression Compared with Mice (A–K) mRNA expression of α ENaC (A), β ENaC (B), and γ ENaC (C) in mouse and rice rat airway cultures. mRNA expression of α ENaC (D), β ENaC (E), and γ ENaC (F) in rice rat airway cultures treated with dexamethasone (DEX) and aldosterone (ALDO). mRNA expression of NR3C1 (G), NR3C2 (H), TMEM16A (I), CFTR (J), and CHRM3 (K). ENaC, epithelial sodium channel; CFTR, cystic fibrosis transmembrane conductance regulator; NR3C1, glucocorticoid receptor; NR3C2, mineralocorticoid receptor; TMEM16, transmembrane member 16A; CHRM3, muscarinic acetylcholine receptor 3. For (A–C) and (G–K), n = 4 cultures from mice (2 female cultures and 2 male cultures representing 6 female and 6 male subjects); n = 4 cultures from the rice rat (2 female cultures and 2 male cultures representing 6 female and 6 male subjects). For (D–F), n = 3 cultures from the rice rat (2 female cultures and 1 male culture representing 6 female and 3 male subjects). *p < 0.05 compared with mouse. All data are shown as mean ± SEM.

We found decreased mRNA expression of α, β, and γ ENaC in the rice rat compared with the mouse (Figures 3A–3C). To examine whether decreased mRNA expression might be due to a steroid hormone requirement, we treated rice rat cultures with aldosterone and dexamethasone using concentrations that augment ENaC expression in primary rat airway cells (Champigny et al., 1994). After 24 h, we re-examined α, β, and γ ENaC mRNA expressions but found no effect (Figures 3D–3F). The lack of an effect was not likely due to a difference in glucocorticoid receptor (NR3C1) or mineralocorticoid receptor (NR3C2) mRNA expression (Figures 3G and 3H). Thus, these data suggested that rice rat did not share the same steroid requirement for ENaC expression as the rat.

We also examined transcript abundance of CFTR and TMEM16A in rice rat and mouse tracheal cultures. We found a robust increase in TMEM16A mRNA expression in the rice rat compared with the mouse (Figure 3I), whereas CFTR mRNA was significantly decreased (Figure 3J). Because carbachol-induced Cl^−^ secretion requires activation of the muscarinic 3 receptor (CHRM3) (Ousingsawat et al., 2009), we also assessed its transcript abundance. No differences in mRNA expression were found (Figure 3K). Thus, these data suggested that increased expression of TMEM16A, but not CHRM3, likely explained the augmented Cl^−^ secretion induced by carbachol.

### Cultured Rice Rat Airway Cultures Secrete Fluid in Response to Carbachol and Display Exaggerated Fluid Expansion in Response to Apical 3.5% NaCl

**Figure 4:**
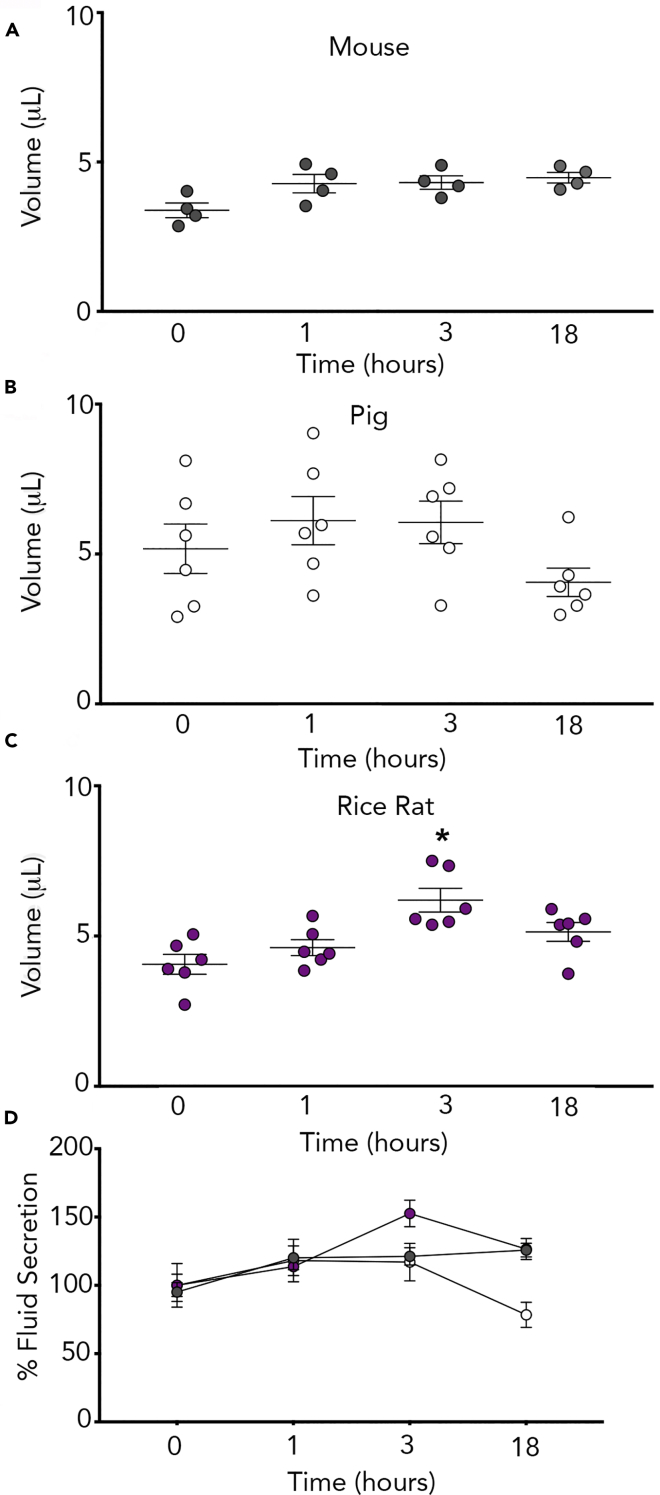
Rice Rat Primary Cultures Secrete Fluid in Response to Carbachol (A–C) Apical fluid secretion in response to 100 μM basolateral carbachol in mouse (A), pig (B), and rice rat (C) airway cultures. (D) Fluid secretion responses normalized to baseline values and expressed as a percent for each species. For all panels, n = 4 cultures from mice (2 female cultures and 2 male cultures representing 6 female and 6 male subjects); n = 6 cultures from the rice rat (3 female cultures and 3 male cultures representing 6 female and 6 male subjects); n = 6 porcine cultures (3 female and 3 male representing 3 individual female and 3 individual male subjects). ∗p < 0.05 compared with baseline. All data are shown as mean ± SEM.

Based upon our ion transport studies, we hypothesized that carbachol would elicit exaggerated fluid secretion in the rice rat airway compared with the piglet or mouse. To test this hypothesis, we measured the apical surface fluid meniscus (see Figures S1A–S1C) using previously published methods (Harvey et al., 2011). Baseline volumes were consistent with previously published values (Figures 4A–4C) (Harvey et al., 2011). In the rice rat, basolateral carbachol significantly increased fluid secretion (Figure 4C). Carbachol did not elicit robust fluid secretion in the piglet (Figure 4B) or mouse (Figure 4A). Normalizing the fluid secretory response to baseline for each species illustrated a qualitatively greater response to carbachol in the rice rat relative to the mouse or pig (Figure 4D). However, lack of a statistically significant interaction (F_6,39_ = 2.1, p = 0.08) precluded any post hoc analyses.

**Figure 5:**
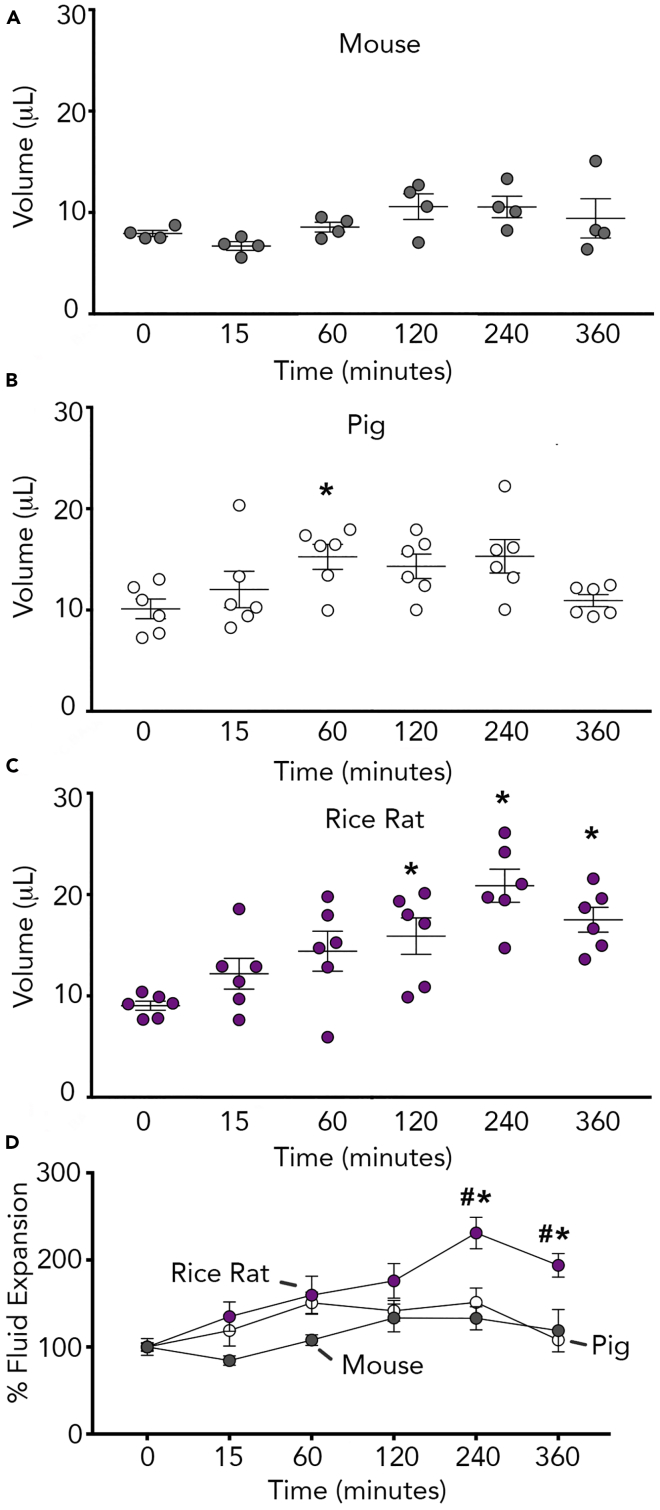
Rice Rat Primary Cultures Display an Exaggerated Fluid Expansion to Hypertonic Saline (A–C) Surface fluid expansion in response to apical 3.5% NaCl increased in in mouse (A), pig (B), and rice rat (C) airway cultures. (D) Fluid expansion responses normalized to baseline values and expressed as a percent for each species. For all panels, n = 4 cultures from mice (2 female cultures and 2 male cultures representing 6 female and 6 male subjects); n = 6 cultures from the rice rat (3 female cultures and 3 male cultures representing 6 female and 6 male subjects); n = 6 porcine cultures (3 female and 3 male representing 3 individual female and 3 individual male subjects). For (B–C) ∗p < 0.05 compared with baseline. For (D) ∗p < 0.05 compared with mouse; #p < 0.05 compared with pig. All data are shown as mean ± SEM.

We also challenged airway cultures with 3.5% hypertonic NaCl to mimic contact with a stimulus that might be encountered in the salt marsh (e.g., sea water aerosol) (Millero et al., 2008) (Figures 5A–5D). In rice rat airway cultures, 3.5% NaCl increased the apical fluid at 2, 4, and 6 h post challenge (Figure 5C). In contrast, apical fluid was only augmented at 1 h in the pig cultures (Figure 5B), whereas the mouse showed insignificant augmentation (Figure 5A). Normalization of the fluid expansion relative to baseline for each species further highlighted greater expansion in the rice rat compared with the mouse or pig (Figure 5D).

## Discussion

We tested the hypothesis that the rice rat airway might have developed unique adaptations for the regulation of ASL. First, we investigated ion transport in rice rat, mouse, and pig airway cultures. We found distinct ion transport properties, including minimal basal short circuit current, decreased amiloride-sensitive current, robust carbachol-mediated current, and minimal F&I-mediated current, in the rice rat airway. These findings suggested greater dependency upon CaCC, which was corroborated by finding increased mRNA expression of TMEM16A and decreased mRNA expression of CFTR. Moreover, we found that carbachol induced apical fluid secretion in rice rat airway cultures, whereas mouse and pig airway cultures were unaffected. Rice rat airway cultures also showed an exaggerated expansion of the ASL in response to 3.5% NaCl compared with the mouse and pig. Thus, these findings support the hypothesis that the rice rat airway exhibits unique ion transport mechanisms compared with animals that do not inhabit salt marshes.

We found minimal basal Isc in the rice rat airway cultures. Low basal Isc has been reported in the colon epithelium of mice with genetic disruption of α ENaC (Barker et al., 1998) and in intestinal segments from mice with targeted disruptions to the *CFTR* gene (Grubb, 1997). Consistent with that, we found limited amiloride-sensitive current and attenuated expression of ENaC in the rice rat airway culture compared with the mouse. These data and others suggest that the rice rat is unique compared with the other rodents. For example, in freshly excised Sprague-Dawley rat tracheas, the amiloride-sensitive current is approximately ∼50–60 μA/cm^2^ (Tuggle et al., 2014), whereas in freshly excised mouse tracheas, it has been reported to be ∼15–20 μA/cm^2^ (Grubb et al., 2001). Epithelial cultures from the Wistar rats show an amiloride-sensitive current that is approximately 2.0 μA/cm^2^ (Hahn et al., 2017), which was nearly three times greater than the average amiloride-sensitive current that we observed in the rice rat cultures (0.59 μA/cm^2^).

Although rice rat tracheal cultures may require steroids for *in vitro* expression of ENaC (Champigny et al., 1994), our findings did not support this hypothesis, as we observed no effect of treatment with aldosterone and dexamethasone on ENaC mRNA expression. This lack of response to mineralocorticoids was reminiscent of pseudohypoaldosteronism in humans (Dirlewanger et al., 2011) and could not be explained by a decrease in mRNA expression of NR3C1 or NR3C2 in rice rat cultures. It is therefore possible that rice rats might offer new insight into pseudohypoaldosteronism.

An additional explanation for the low amiloride-sensitive current in the rice rat is naturally occurring mutations that impact ENaC trafficking and/or function. Indeed, α ENaCa, a naturally occurring truncated isoform of α ENaC, fails to generate amiloride-sensitive current when expressed in *Xenopus* oocytes (Li et al., 1995). We are cloning rice rat ENaC subunits to examine this possibility. If true, then it is conceivable that Na^+^ is absorbed through an ENaC-independent mechanism (O'Brodovich et al., 2008).

The secretion of Cl^−^ in response to carbachol was greater in the rice rat compared with the pig and mouse. Based on published studies, the degree of carbachol-mediated Cl^−^ secretion in the rice rat is also greater than expected for the rat. Specifically, in tracheal cultures from Wistar rats, the amount of carbachol-mediated Cl^−^ secretion is 8.5 μA/cm^2^ (Hahn et al., 2017), which is approximately half of what we observed in the rice rat (19.7 μA/cm^2^). Consistent with a greater response to carbachol, rice rat cultures also showed elevated expression of TMEM16A mRNA compared with mouse. Thus, it is likely that enhanced TMEM16A expression contributes to the enhanced carbachol-mediated Cl^−^ secretion in rice rat. It is also possible that other channels contribute (Rock et al., 2009).

We found that Cl^−^ secretion through CFTR was significantly decreased in the rice rat compared with the pig, and qualitatively less than the mouse. Paralleling this finding, mRNA expression for CFTR was also decreased in rice rat cultures compared with mouse cultures. This diminished CFTR activity appears to have minimal negative effects in the rice rat airway, as they show no overt signs of lung disease, even as carriers of hantavirus (Holsomback et al., 2013). In CFTR knockout mice, and in humans and rats with CFTR mutations, TMEM16A is thought to compensate for loss of CFTR (Billet and Hanrahan, 2013, Clarke and Boucher, 1992, Tuggle et al., 2014). Ergo, it is likely that TMEM16A also compensates in rice rats.

Previous studies have shown that epithelia from saline-adapted animals have decreased Na^+^ absorption and increased Cl^−^ secretion (Foskett et al., 1981; Kultz and Onken, 1993). If we interpret our data to indicate that the rice rat airway has evolved to also absorb less Na^+^ through ENaC and secrete more of Cl^−^ through CaCC, of what advantage might this be? We speculate that the rice rat airway when exposed to extreme heat (Silliman, 2014) and aerosols containing high NaCl concentrations (Gabriel et al., 2002) experiences a transient increased ASL tonicity. This increase in ASL tonicity activates the vagus nerve to elicit cholinergically mediated Cl^−^ secretion (Wine, 2007), which acutely increases water on the airway surface. Simultaneously, the decreased Na^+^ absorption through ENaC and limited Cl^−^ absorption through CFTR ensure that the ASL expansion in response to increased tonicity is sustained. Combined, these processes likely help protect the airway against dehydration and ensure proper mucociliary transport (Matalon et al., 2015). However, having too much water on the surface of the airway is also of negative consequence as newborn mice lacking α ENaC die due to an inability to clear fluid out of the lungs (Hummler et al., 1996). Similarly, improper airway fluid absorption is observed in patients with pseudohypoaldosteronism who have mutations in ENaC (Kala Ahluwalia et al., 2014, Kerem et al., 1999). Although we have no evidence to suggest that rice rats have difficulty with airway fluid absorption under basal conditions, we recently reported that atropine, a cholinergic antagonist, was required for proper respiratory patency during specific anesthesia regimens in the rice rat (Jiron et al., 2019). If excess airway fluid is produced, then the rice rat might clear those secretions through enhanced mucociliary transport (Kerem et al., 1999) and swallowing. Thus the unique airway transport of the rice rat might be advantageous in the salt marsh, but potentially disadvantageous in other environments.

### Limitations of the Study

Although the *in vitro* system used here confers its own advantages, *including morphology* (Karp et al., 2002), transcriptional profiles (Pezzulo et al., 2011), and ion transport properties similar to that observed *in vivo* (Itani et al., 2011), *in vivo* validation would be required to conclusively support the findings of this study. Second, it is also worth noting that we did not investigate other epithelia in other tissues. The rice rat is an emerging research model used to study spontaneous periodontitis and medication-related osteonecrosis of the jaw (Aguirre et al., 2012, Messer et al., 2018, Messer et al., 2019). The interaction of the gingival epithelia with oral microbiota influences the status of health or disease (Tribble and Lamont, 2010). Although we did not investigate non-airway epithelia, it is interesting to consider that ion transport properties of other epithelia, such as the gingival epithelia, might also be distinct or modified compared with those of other animals. If so, then perhaps those properties contribute to the well-described predilection of periodontitis in the rice rat (Aguirre et al., 2017, Gotcher and Jee, 1981a, Gotcher and Jee, 1981b, Gupta and Shaw, 1956a, Gupta and Shaw, 1956b).

In summary, our findings suggest that the rice rat airway has distinct ion transport properties that likely facilitate survival in the marsh salt environment. Perhaps examining the airway physiology of additional mammals that inhabit extreme environments might reveal similar, or perhaps even dissimilar, discoveries.

## Methods

All methods can be found in the accompanying Transparent Methods supplemental file.

## Supplemental Information

Document S1. Transparent Methods and Figure S1
