# Supplementary material for: Attenuated Amiloride-Sensitive Current and Augmented Calcium-Activated Chloride Current in Marsh Rice Rat (Oryzomys palustris) Airways
Source: iScience. 2019 Aug 8;19:737–48. doi: 10.1016/j.isci.2019.08.011 (PMC6731178; doi:10.1016/j.isci.2019.08.011)
Supplement: Document S1. Transparent Methods and Figure S1 [file mmc1.pdf]

ISCI, Volume *19*

Supplemental Information

Attenuated Amiloride-Sensitive Current and Augmented Calcium-Activated Chloride Current in Marsh Rice Rat (*Oryzomys palustris*) Airways

Shin-Ping Kuan, Yan-Shin J. Liao, Katelyn M. Davis, Jonathan G. Messer, Jasenka Zubcevic, J. Ignacio Aguirre, and Leah R. Reznikov

Supplemental Information

Supplemental Data and Figure Legends

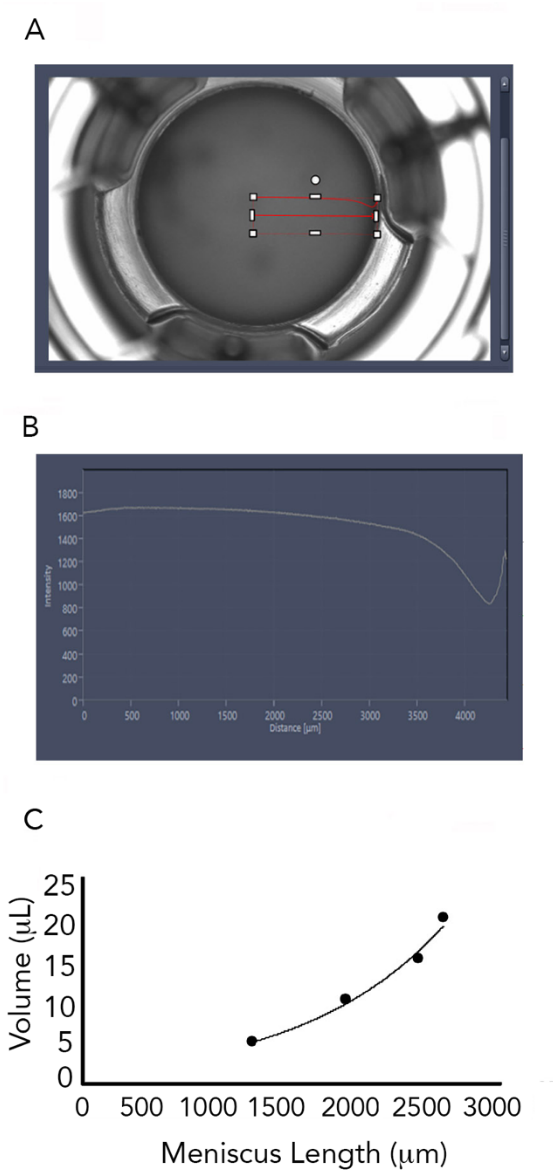

Figure S1. Measuring fluid secretion using reflected light and measurement of meniscus length. Related to Figure 4 and Figure 5.

(A) Representative airway culture with intensity profile highlighted corresponding to red arrow shown directly above as red line with a “valley”. (B) Closer view of the red trace in panel A showing the XY coordinates corresponding to profile intensity (Y) and length

(X). The length of the fluid meniscus was calculated as the distance between the greatest decrease in pixel intensity (at cell culture insert edge, shown on the right) minus the average baseline intensity taken from the middle of the culture. (C) Standard curve generated from 2 separate cultures when known volumes of fluid were added to the apical surface and the meniscus length measured. An exponential curve was fitted with a R^2 value of 0.99 resulting in the following equation: y = 1.5373e^0.001x. This equation was used to extrapolate volume based upon meniscus length.

Transparent Methods.

Animal.

Young male and female Landrace piglets (1 week old) (n = 5 female, n = 4 males) were purchased from an outside vendor and euthanized with intravenous euthasol (Henry Schein, Virbac) (Reznikov et al., 2013). Adult male (n =18) and female (n= 22) rice rats were derived from an in-house breeding colony (Aguirre et al., 2015) and euthanized by CO_2 inhalation followed by cervical dislocation. Similarly, adult male (n = 8) and female C57BL/6 mice (n = 8) were obtained from an in-house breeding colony and euthanized by CO_2 inhalation followed by cervical dislocation and bilateral thoracotomy. All procedures were approved by the University of Florida IACUC and the University of Florida Animal Care Services, which is an AAALAC-accredited animal care and use program.

Primary cultures of differentiated airway epithelia. Epithelial cells were isolated from piglet tracheas by enzymatic digestion (Pronase, Roche; DNASE, Sigma) seeded onto

collagen (Corning Collagen I, Rat Tail)-coated permeable filter supports (Corning Transwell polycarbonate membrane inserts, area = 0.33 cm^2, pore size = 4 µM), and grown at the air-liquid interface using previously described procedures (Karp et al., 2002). Differentiated epithelia were studied at a minimum of 14 days after seeding. For the rice rat and mouse, 2-3 tracheas were pooled to isolate an adequate amount of cells for 3-4 permeable filter supports. For the pig, a single trachea yielded on average 24 inserts. Isolation procedures for rice rat and mouse airway epithelial cells were similar to those established for the piglet, except digestion of the tracheas occurred for only ~24-30 hours for rice rat and mouse tracheas (compared to 48 hours for the piglet).

Electrophysiological measurements of cultured airway epithelia. Cultured epithelia were studied in modified Ussing chambers (EasyMount Ussing Chamber System; Physiologic Instruments). Transepithelial voltage was maintained at 0 mV and short-circuit current (Isc) measured (VCC MC-8; Physiologic Instruments). Isc and delta (Δ) Isc were reported.

Cultured airway epithelia were bathed on the apical and basolateral surfaces with the following solution: 135 mM NaCl, 2.4 mM K_2HPO_4, 0.6 mM KH_2PO_4, 1.2 mM $CaCl_2$, 1.2 mM $MgCl_2$, 10 mM dextrose, 5 mM HEPES, pH 7.4 (NaOH). The solution was maintained at 37°C and gassed with compressed air (Chen et al., 2010; Stoltz et al., 2013).

The following protocol was performed: (a) measurements at baseline; (b) apical 100 µM amiloride to inhibit ENaC; (c) 100 µM basolateral carbachol to simulate CaCC (d) 30 µM

CaCCinh-A01 to inhibit CaCC (He et al., 2011); (e) apical 10 µM forskolin and 100 µM IBMX to increase cAMP and activate CFTR; (f) apical 100 µM GlyH-101 to inhibit CFTR. All drug concentrations were selected according to our previous studies (Chen et al., 2010; Stoltz et al., 2013).

Drugs used for Ussing chamber studies and cell culture. Ussing chamber studies: amiloride hydrochloride hydrate (Sigma); carbamylcholine chloride (carbachol) (Sigma); forskolin (Sigma); 3-isobutyl-1-methylxanthine (Sigma); Glyh-101 (a gift from the Cystic Fibrosis Foundation Therapeutics and Robert Bridges, Rosalind Franklin University of Medicine and Science, North Chicago, Illinois, USA); and CaCCinh-A01 (Sigma). Cell culture antibiotics (concentrations used are taken from reference (Karp et al., 2002): amphotericin B (Sigma); gentamicin (Gibco); penicillin-streptomycin (Gibco). Additional antibiotics/antifungals used include: tobramycin (Alfa Aesar), final concentration 25 µg/ml; fluconazole (Selleckchem), final concentration 2 µg/ml; piperacillin sodium (Selleckchem), final concentration 17.8 µg/ml; tazobactam (Selleckchem), final concentration 2.2 µg/ml. For examining a potential requirement of steroid hormones for expression ENaC, aldosterone (ACROS) was dissolved in 100% DMSO for a stock concentration of 1 mM. Stocks were diluted 1:1,000 in cell culture media for a final concentration of 1 µM. This dose was selected because it has been shown to increase ENaC expression in primary rat airway epithelial cells (Champigny et al., 1994). Dexamethasone (Fisher Scientific) was also dissolved in 100% DMSO to a stock concentration of 100 µM. Stocks were diluted 1:1,000 in cell culture media for a final concentration of 100 nM. Again, the dose was selected because it has been shown to

increase ENaC expression in primary rat airway epithelial cells (Champigny et al., 1994). Cultures were incubated in drugs for 24 hours prior to harvest and examination of mRNA levels. This time point was selected based upon previous studies (Champigny et al., 1994; Sayegh et al., 1999).

Immunofluorescence. Primary culture epithelial cells were fixed in 4% paraformaldehyde for 15 minutes. Following fixation, cells were washed three times for 10 minutes PBS and permeabilized for 15 minutes in 0.15% Triton X-100 (Fisher Scientific)/PBS solution. Cells were then blocked in a 4% normal goat serum (Jackson Immuno Research Labs)/Superblock buffer in PBS (ThermoFisher Scientific) for 60 minutes. Cells were incubated overnight at room temperature in primary antibodies. Following overnight incubation, cells were washed, incubated in appropriate secondary antibodies (listed below), and washed again. A Hoechst 33343 (ThermoFisher Scientific) stain was performed to identify nuclei. Cells were mounted in Vectashield Hardset (Vector labs) and cover slipped. Images were captured on a Zeiss Axio Zoom V16. Identical microscope settings for assessment of a single marker across species.

Antibodies. Rabbit anti-ZO-1, 1:500 (Life Technologies, 617300); mouse anti-acetyl alpha tubulin, clone 6-11B-1, 1:500 (EMD Millipore, MABT868). The antigen for the ZO-1 antibody corresponds to residues 463-1109 of human ZO-1. This antibody has been used previously to identify ZO-1 in multiple species, including rat (Bordin et al., 2004) and human (Excoffon et al., 2009). According to the manufacturer, the antigen for the

acetyl alpha tubulin is 15 S dynein fraction from the sea urchin sperm axoneme. This antibody has been used to identify human, *Drosophila*, and *Chlamydomonas* acetylated alpha tubulin (Piperno and Fuller, 1985). Secondary antibodies were used at 1:1000 and included: goat anti-mouse IgG (H+L) dylight 488 (ThermoFisher Scientific, 35505); alexa fluor 488 goat anti-rabbit IgG (H+L) (ThermoFisher Scientific, A11034).

Fluid secretion studies. We implemented the reflected light microscopy method to measure the meniscus of the secreted fluid (Harvey et al., 2011). Briefly, cultures were placed on a heated stage maintained at 37°C and imaged using a Zeiss Axio Zoom V16. The following buffer bathed the basolateral side: 135 mM NaCl, 2.4 mM K_2HPO_4, 0.6 mM KH_2PO_4, 1.2 mM $CaCl_2$, 1.2 mM $MgCl_2$, 10 mM dextrose, 5 mM HEPES, pH 7.4 (NaOH). Light intensity and magnification were held constant for all cultures. A single image was taken at designated time points. Cultures were returned to the incubator in between imaging. The intensity profile of the image was examined using Zen Pro software analysis (Zeiss). The XY coordinates of the intensity profile correlated to distance (X) and intensity (Y). The length of the fluid meniscus could therefore be readily identified as the distance between the greatest decrease in pixel intensity (at cell culture insert edge) minus the average baseline intensity (in the middle of the culture). A 4-point regressive curve used to convert meniscus lengths to volumes. The curve was generated by adding known volumes (5, 10, 15, 20 µl) of the bathing fluid were added to the apical surface of two separate cultures (e.g., duplicate standard curves) plotting regression curve (R^2 value of 0.99: y = 1.5373e^0.001x). Apical fluid secretion to 100

μM basolateral carbachol was measured. The average salinity of sea water is 3.5% (Millero et al., 2008). Therefore, we also measured fluid secretion in response to 5 μl of apical 3.5% hypertonic saline (Harvey et al., 2011).

RNA isolation, cloning and qRT-PCR. RNA from rice rat and mouse whole trachea, as well as airway epithelial cultures from rice rats and mice, were isolated using methods previously described (Reznikov et al., 2019). Briefly, a RNeasy Lipid Tissue kit (Qiagen) with optional DNase digestion (Qiagen) was used to isolate RNA. The RNA concentrations were assessed using a NanoDrop spectrophotometer (ThermoFisher Scientific). RNA was reverse transcribed for trachea tissues (500 ng) and cultures (50 ng) using Superscript VILO Master Mix (ThermoFisher) (Reznikov et al., 2019). RNA and master mix were incubated for 10 mins at 25°C, followed by 60 mins at 42°C, followed by 5 mins at 85°C.

Small segments of mRNA for rice rat CFTR, TMEM16A, α ENaC, β ENaC, γ ENaC, muscarinic 3 receptor (CHRM3), glucocorticoid receptor (NR3C1), mineralocorticoid receptor (NR3C2) and RPL13a were amplified using primers designed to mouse orthologs from both cultured epithelia and tracheal epithelia (Table 1). Primers were ordered through IDT (Coralville, IA). Amplification was achieved using Platinum Taq High Fidelity DNA polymerase (ThermoFisher) and 100 ng of cDNA. Cycling parameters consisted of 35 cycles at 94 °C for 15 s, 52 °C for 20 s, and 68 °C for 30 s, and a final extension cycle of 3 minutes. Amplicons were run on a 1.5% agarose gel, extracted, and purified using a gel extraction kit (Qiagen). Amplicons were the sequenced using

GENEWZ services (South Plainfield, NJ). Sequences were BLAST in NCBI and areas of 100% homology between rice rat and mouse were identified. Primers were designed according to areas exhibiting 100% homology to enable amplification of both mouse and rice rat genes with same primers (Table 2) and ordered through IDT (Coralville, IA). Transcripts for CFTR, TMEM16A, α ENaC, β ENaC, γ ENaC, CHRM3, NR3C1 and NR3C2 were quantified with qRT-PCR using RPL13a as a reference gene. All qRT-PCR data were acquired using fast SYBR green master mix (Applied Biosystems) and a LightCycler 96 (Roche). Standard ΔΔCT methods were used for analysis. SYBR green dissociation curves revealed the presence of single amplicons for each primer pair.

Data and Code Availability. The accession number for the rice rat sequences reported in this manuscript are: GenBank: MN200425; GenBank: MN200426; GenBank: MN200427; GenBank: MN200428; GenBank: MN200429; GenBank: MN200430; GenBank: MN200431; GenBank: MN200432; GenBank: MN200433.

Statistical Analysis. A one-way ANOVA was used to examine differences in ion transport. Post hoc comparisons between mouse and rice rat or pig and rice rat were achieved using Dunnett's multiple comparisons test. A one-way ANOVA with repeated measures was performed to assess fluid secretion in mouse, rice rat or pig airway cultures. Post hoc comparisons were achieved using Dunnett's multiple comparisons test for each species relative to baseline measures. For normalized secretion responses, a two-way ANOVA was performed with time as a repeated measure. When a significant interaction was observed, a Tukey's multiple comparisons test was

performed. Transcript abundances were assessed using a two-tailed unpaired student test (for each gene, mouse versus rice rat). Values with $p < 0.05$ were considered statistically significant. All analyses were performed in GraphPad Prism 7.0a.

Aguirre, J.I., Edmonds, K., Zamora, B., Pingel, J., Thomas, L., Cancel, D., Schneider, L., Reinhard, M.K., Battles, A.H., Akhter, M.P., *et al.* (2015). Breeding, husbandry, veterinary care, and hematology of marsh rice rats (Oryzomys palustris), a small animal model for periodontitis. J Am Assoc Lab Anim Sci *54*, 51-58.

Bordin, M., D'Atri, F., Guillemot, L., and Citi, S. (2004). Histone deacetylase inhibitors up-regulate the expression of tight junction proteins. Mol Cancer Res *2*, 692-701.

Champigny, G., Voilley, N., Lingueglia, E., Friend, V., Barbry, P., and Lazdunski, M. (1994). Regulation of expression of the lung amiloride-sensitive Na+ channel by steroid hormones. EMBO J *13*, 2177-2181.

Chen, J.H., Stoltz, D.A., Karp, P.H., Ernst, S.E., Pezzulo, A.A., Moninger, T.O., Rector, M.V., Reznikov, L.R., Launspach, J.L., Chaloner, K., *et al.* (2010). Loss of anion transport without increased sodium absorption characterizes newborn porcine cystic fibrosis airway epithelia. Cell *143*, 911-923.

Excoffon, K.J., Koerber, J.T., Dickey, D.D., Murtha, M., Keshavjee, S., Kaspar, B.K., Zabner, J., and Schaffer, D.V. (2009). Directed evolution of adeno-associated virus to an infectious respiratory virus. Proc Natl Acad Sci U S A *106*, 3865-3870.

Harvey, P.R., Tarran, R., Garoff, S., and Myerburg, M.M. (2011). Measurement of the airway surface liquid volume with simple light refraction microscopy. Am J Respir Cell Mol Biol *45*, 592-599.

He, Q., Halm, S.T., Zhang, J., and Halm, D.R. (2011). Activation of the basolateral membrane Cl- conductance essential for electrogenic K+ secretion suppresses electrogenic Cl- secretion. Exp Physiol *96*, 305-316.

Karp, P.H., Moninger, T.O., Weber, S.P., Nesselhauf, T.S., Launspach, J.L., Zabner, J., and Welsh, M.J. (2002). An in vitro model of differentiated human airway epithelia. Methods for establishing primary cultures. Methods Mol Biol *188*, 115-137.

Millero, F.J., Feistel, R., Wright, D.G., and McDougall, T.J. (2008). The composition of Standard Seawater and the definition of the Reference-Composition Salinity Scale. Deep-Sea Res Pt I *55*, 50-72.

Piperno, G., and Fuller, M.T. (1985). Monoclonal antibodies specific for an acetylated form of alpha-tubulin recognize the antigen in cilia and flagella from a variety of organisms. J Cell Biol *101*, 2085-2094.

Reznikov, L.R., Dong, Q., Chen, J.H., Moninger, T.O., Park, J.M., Zhang, Y., Du, J., Hildebrand, M.S., Smith, R.J., Randak, C.O., *et al.* (2013). CFTR-deficient pigs display peripheral nervous system defects at birth. Proc Natl Acad Sci U S A *110*, 3083-3088.

Reznikov, L.R., Liao, Y.S.J., Gu, T., Davis, K.M., Kuan, S.P., Atanasova, K.R., Dadural, J.S., Collins, E.N., Guevara, M.V., and Vogt, K. (2019). Sex-specific airway

hyperreactivity and sex-specific transcriptome remodeling in neonatal piglets challenged with intra-airway acid. Am J Physiol Lung Cell Mol Physiol *316*, L131-L143.
Sayegh, R., Auerbach, S.D., Li, X., Loftus, R.W., Husted, R.F., Stokes, J.B., and Thomas, C.P. (1999). Glucocorticoid induction of epithelial sodium channel expression in lung and renal epithelia occurs via trans-activation of a hormone response element in the 5'-flanking region of the human epithelial sodium channel alpha subunit gene. J Biol Chem *274*, 12431-12437.
Stoltz, D.A., Rokhlina, T., Ernst, S.E., Pezzulo, A.A., Ostedgaard, L.S., Karp, P.H., Samuel, M.S., Reznikov, L.R., Rector, M.V., Gansemer, N.D., *et al.* (2013). Intestinal CFTR expression alleviates meconium ileus in cystic fibrosis pigs. J Clin Invest *123*, 2685-2693.
